# The Role of Astaxanthin as a Nutraceutical in Health and Age-Related Conditions

**DOI:** 10.3390/molecules27217167

**Published:** 2022-10-23

**Authors:** Geir Bjørklund, Amin Gasmi, Larysa Lenchyk, Mariia Shanaida, Saba Zafar, Pavan Kumar Mujawdiya, Roman Lysiuk, Halyna Antonyak, Sadaf Noor, Muhammad Akram, Kateryna Smetanina, Salva Piscopo, Taras Upyr, Massimiliano Peana

**Affiliations:** 1 Council for Nutritional and Environmental Medicine (CONEM), Toften 24, 8610 Mo i Rana, Norway; 2 Société Francophone de Nutrithérapie et de Nutrigénétique Appliquée, 69100 Villeurbanne, France; 3 Institute for Advanced Training of Pharmacy Specialist, National University of Pharmacy, 61001 Kharkiv, Ukraine; 4 CONEM Ukraine Pharmacognosy and Natural Product Chemistry Research Group, National University of Pharmacy, 61002 Kharkiv, Ukraine; 5 Department of Pharmacognosy and Medical Botany, I. Horbachevsky Ternopil National Medical University, 46001 Ternopil, Ukraine; 6 Department of Biochemistry and Biotechnology, The Women University, Multan 60000, Pakistan; 7 Inochi Care Private Limited, Main Market, Malviya Nagar, New Delhi 110017, India; 8 Department of Pharmacognosy and Botany, Danylo Halytsky Lviv National Medical University, 79010 Lviv, Ukraine; 9 CONEM Ukraine Life Science Research Group, Danylo Halytsky Lviv National Medical University, 79010 Lviv, Ukraine; 10 Department of Ecology, Ivan Franko National University of Lviv, 79005 Lviv, Ukraine; 11 Institute of Molecular Biology and Biotechnology, Bahauddin Zakariya University, Multan 60800, Pakistan; 12 Medicine, Government College University, Faisalabad 38000, Pakistan; 13 Department of Organic Chemistry and Pharmacy, Lesya Ukrainka Volyn National University, 43025 Lutsk, Ukraine; 14 Department of Pharmacognosy, National University of Pharmacy, 61168 Kharkiv, Ukraine; 15 Department of Chemical, Physical, Mathematical and Natural Sciences, University of Sassari, 07100 Sassari, Italy

**Keywords:** astaxanthin, health benefit, human aging, reactive oxygen species, antioxidant supplementation, neuroprotection, skin protection, carotenoid

## Abstract

The current review provides an up-to-date analysis of scientific data on astaxanthin (ASX) sources and experimental studies on its health benefits as a potent antioxidant in the aging process. ASX is a liposoluble carotenoid nutrient and reddish-orange pigment, naturally synthesized by numerous microalgae, yeasts, and bacteria as secondary metabolites. Provides a reddish hue to redfish and shellfish flesh that feed on ASX-producing microorganisms. The microalga *Haematococcus pluvialis* is the most important source for its industrial bioproduction. Due to its strong antioxidant properties, numerous investigations reported that natural ASX is a more significant antioxidant agent than other antioxidants, such as vitamin C, vitamin E, and β-carotene. Furthermore, several data show that ASX possesses important nutraceutical applications and health benefits, especially in healthy aging processes. However, further studies are needed for a deeper understanding of the potential mechanisms through which ASX could lead to its effective role in the healthy aging process, such as supporting brain health and skin homeostasis. This review highlights the current investigations on the effective role of ASX in oxidative stress, aging mechanisms, skin physiology, and central nervous system functioning, and shows the potential clinical implications related to its consumption.

## 1. Introduction

Natural compounds can display therapeutic effects against various chronic conditions, from inflammation to cancer [1,2]. Nutraceuticals are food components that have both nutritional and medicinal properties, and have been used since 1980 as an essential part of complete wellness and health [3,4]. Nutraceuticals can be extracted from plants, fungi, bacteria, or animal products, then concentrated and administered in a suitable pharmaceutical dosage form to prevent or treat some human pathological conditions [2].

Astaxanthin (ASX) is a liposoluble carotenoid and a reddish-orange pigment. ASX plays a role in circulating lipoproteins and cell membranes, and has significant antioxidant and anti-inflammatory activity [5,6]. It can be naturally synthesized by numerous microalgae, yeasts, and bacteria as a secondary metabolite. It supplies a reddish hue to the to redfish, sea bream and salmon flesh and crustaceans (crabs and shrimps) that feed on ASX-producing microbes [7,8]. The microalga *Haematococcus pluvialis* is considered the most important source of its industrial biological production [7,9,10]. However, commercial production of ASX has traditionally been done by chemical synthesis [11]. 

ASX is also used commercially for feed production. As salmon cannot naturally synthesize ASX, the salmon grown on farms fail to develop the characteristic flesh color of their wild counterparts. ASX is used together with canthaxanthin in feed to dye salmon flesh, as well as for trout, and shrimp [12]. Likewise, ASX gives the characteristic color to egg yolk and broiler chicken carcass. 

Research carried out on ASX shows that it helps reduce the negative effects of aging by neutralizing reactive oxygen species (ROS) and reactive nitrogen species (RNS) within the body’s cells which lead to overloading of systems. defense and the consequent oxidative damage [13,14]. Recently, ASX was found to have a several times greater effect than that of *β*-carotene on singlet oxygen quenching, and an antioxidant function of up to 100 times more significant than vitamin E against lipid peroxidation [15]. ASX is an antioxidant and anticancer agent that prevents cardiovascular diseases, diabetes, and neurodegenerative disorders, and stimulates the immune system [14,16,17,18,19,20,21]. The anti-aging role of ASX has been attributed to its antioxidant and anti-inflammatory properties, preventing age-related muscle deterioration and improving energy generation in the mitochondria [22,23]. ASX can help eliminate free radicals produced during exercise and aerobic metabolism in muscles [24]. It can also help boost immunity, resist fatigue, and delay aging [25].

The global consumption of many sources of ASX in food and nutraceuticals continues to increase. ASX products are used for pharmaceutical applications in dosage forms, such as tablets, capsules, soft gels, creams, oils, biomass, and granulated powders [26]. Due to its beneficial properties, ASX can also be used as a food colorant and an antioxidant to improve foods’ nutritional value and sensory quality. The European Commission considers ASX a food dye signed as E161 [27]. In 2019, the average daily intake (ADI) was set at 0.2 mg/day by the Additives and Products or Substances used in Animal Feed (FEEDAP) committee of the European Food Safety Authority (EFSA) [28].

The potential pharmacological effects of ASX, which include anti-inflammatory and antioxidant activities, as well as neuro and skin-protective effects, have brought it to public attention. Previous studies have demonstrated that, due to its better biosafety and high bioavailability, ASX has strong therapeutic potential against many diseases, such as in modulating neuroinflammation [29], ocular diseases [30], cardiovascular, aging, neurodegenerative, respiratory, and liver disease [31]. In this study, we summarize the therapeutic impact of ASX in controlling oxidative stress related to the aging process, skin conditions, and the effects on the central nervous system.

## 2. Structure and Sources of Astaxanthin

**Figure 1:**
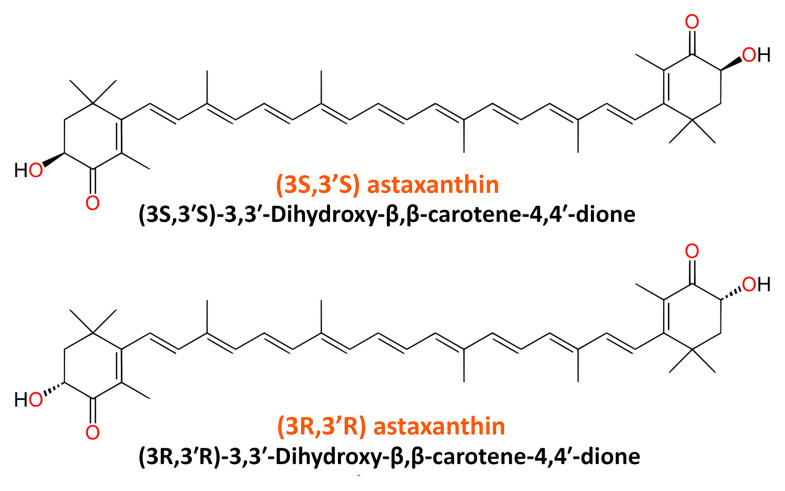
Chemical structure of two natural enantiomers of astaxanthin.

ASX belongs to a family of naturally occurring organic pigments called carotenoids. It is a keto-carotenoid classified as a xanthophyll with the chemical IUPAC’s name 3,3′-dihydroxy-β, β-carotene-4,4′-dione. In nature, ASX molecules exist as stereoisomers, geometric isomers, and in free and esterified forms, among which the most abundant are (3S, 3′S) and (3R 3′R) stereoisomers. The empirical formula of ASX is C_40_H_52_O_4_. The structures of two natural enantiomers of ASX are shown in Figure 1.

ASX is produced by several marine and freshwater microorganisms, including microalgae, fungi, lichens, and bacteria (Table 1). It can also be obtained from redfish and crustaceans that feed on ASX-producing microorganisms [32].

Microalgae and yeasts are very important commercial sources of natural ASX. Microalgae are already gaining popularity as a source of food, which makes their implementation in the food system easier [42]. The microalgae *Haematococcus pluvialis* synthesizes the highest amount of ASX in nature, which makes it an optimal choice for the commercial production of ASX. *Haematococcus pluvialis* may contain up to 3.8% of ASX on a dry weight basis. A naturally cultivated form of ASX is produced from the microalgae *Haematococcus pluvialis*. ASX is also produced by algal species, such as *Neochloris wimmeri, Enteromorpha intestinalis, Chlorococcum,* *Chromochloris zofingiensis,* etc. [35]. Hence, using algal-based ASX could help overcome its chemical production.

Aerobic Gram-negative bacterium *Paracoccus carotinifaciens sp. nov.* and basidiomycetous yeast *Xanthophyllomyces dendrorhous (syn.* *Phaffia rhodozyma*) also produce high-value carotenoid and are used for ASX production at the industrial scale. High-titer production of ASX by the semi-industrial fermentation of *Xanthophyllomyces dendrorhous* could be easily scaled-up to an industrial application for producing this xanthophyll [43]. Yeast *Phaffia rhodozyma* was first isolated from the exudates of trees in mountainous regions of Japan and Alaska during the early 1970s [44]. The yeast was recognized as very special because due to its ability to synthesize ASX. Later, *Phaffia rhodozyma* was approved by European Commission for use as a pigment source in feeding stuff for salmon and trout [45]. Several species of Lichens are also producing ASX, although not in industrially exploitable quantities [46].

The content of ASX found in salmon were recorded in the range of 26–38 mg/kg in the flesh of wild salmon (*Oncorhynchus* species) and 6–8 mg/kg in the flesh of farmed Atlantic salmon, which is a reason for consumers’ preferences for wild salmon over farmed salmon. Big trout may contain 6 to 25 mg/kg of ASX in the flesh [47]. Recent findings are associated with the possible use of crustacean by-products as a source of natural ASX [10]. Shrimp, crabs, krill, crayfish, and copepods can also serve as a source of ASX [48]. 

ASX is also produced through chemical synthesis and genetic engineering. In the second half of the 20th century, ASX was successfully synthesized from isophorone, *cis*-3-methyl-2-penten-4-yn-1-ol, and a symmetrical C_10_-dialdehyde. Although the production by chemical synthesis is a more cost-effective way to obtain ASX, this process does not promise to give a pure chemical compound, but a combination of different isoforms, some of which cannot be found in nature; thus, synthetic or chemical ASX molecules have different activities than their natural counterparts [12,49,50,51]. The biological effect of natural ASX is much stronger than its synthetic analog, which may be due to the presence of all three isomers in synthetic ASX: two enantiomers (3’R 3R and 3’S 3S) and one mesoform (3R 3’S), while natural ASX only has 3S, 3′S or 3R, 3′R (Figure 1) [52]. Around 95% of natural ASX is mono- or di-esterified with fatty acid molecules, while synthetic ASX is free.

Studies demonstrate that natural ASX is 6000 times more powerful than vitamin C, 770 times more active than coenzyme Q10 (CoQ_10_), 100 times more potent than vitamin E, and five times more powerful than β-carotene in trapping energy from singlet oxygen, one of the most common ROS found in the human body [53,54,55]. Interestingly, ASX neutralizes ROS by either donating or accepting electrons without being damaged in this process [56]. Its distinct chemical characteristics make it unique when compared to other antioxidants.Even though their structures are similar, ASX contains 13 conjugated double bonds, while β-carotene only has 11. Oxo groups are located in the fourth and fourth prime locations in the cyclohexene structure. The length of the electron-rich, conjugated double bonds determines the antioxidant potential of carotenoids. When compared to β-carotene and vitamin E, ASX has greater efficacy due to an extension of the conjugated double bond system [57]. The molecule of ASX is relatively polar due to the presence of hydroxyl groups in positions 3 and 3’. While nonpolar carotenoids, including β-carotene and lycopene, are present between the lipid bilayer of membranes and may cause the phospholipid molecules’ intermolecular packing to become disrupted [58,59], ASX with polar end groups that extend toward the head group areas of the lipid bilayer can cross the membrane. The structure of the lipids that make up the membrane remains preserved [5,58,59]. As a consequence, ASX halts free radical chain reactions and scavenges lipid peroxyl radicals to function as a chain-breaking antioxidant. ASX crossing the cell lipid bilayer membrane, allows its terminal rings to efficiently scavenge ROS on the membrane surface while its polyene chain traps ROS inside the membrane [5]. Consequently, ASX acts as a scavenger of free radicals in the inner membrane layer and simultaneously could control oxidation on the membrane surface.

ASX also acts as a powerful antioxidant without having any pro-oxidative properties [60]. As a result, ASX effectively neutralizes destructive ROS while being gentle on the body’s cells. Thus, its unique structure and flexibility to neutralize free-radicals from both hydrophilic and hydrophobic boundaries of a cell membrane make it a stronger antioxidant compared to all other antioxidants, such as β-carotene, vitamin E, etc. ASX counteracts potentially harmful free radicals/ROS by trapping energy (quenching) and through the transfer of electrons or hydrogen abstraction (scavenging) [56].

The world market of ASX exceeds USD 400 million per year, and the share of natural ASX on it is about one percent, while its market value is 2–7 times higher than that of the synthetic analog [61]. According to experts, ASX production worldwide will reach 190 tons by 2024. Synthetic ASX is mainly used for aquaculture [61]. Therefore, the urgent task is to increase the production of natural ASX, especially due to the high market demand. 

An alternative to chemical synthesis is the genetic modification of microorganisms and an increase in the amount of ASX they produce. Several methods are being developed for this purpose. Thus, using the genetically modified organisms (GMO) *Yarrowia lipolytica, Escherichia coli,* and *Saccharomyces cerevisiae,* an enhancement in the synthesis of ASX to 8–10 mg/g DCW occurred [38,62,63]. However, the commercial application of these genetically modified microorganisms and the use of their products in food or feed will need to undergo special regulations to ensure their safety. Studies whose purpose was to establish ways to increase the synthesis of ASX by microorganisms without changing their genetic code, as well as effective methods for extraction and delivery systems, have also been conducted [64]. It was also found that the synthesis of ASX is promoted by a decrease in the content of nitrates and phosphates in the medium and an increase in the amount of ferrous iron and sodium chloride, as well as an increase in light exposure [25,65]. The biotechnological productions of ASX and other carotenoids by yeasts or *Escherichia coli* have been described by researchers [38]. Astaxanthin productivity in engineered *Chlamydomonas reinhardtii* has been evaluated by Perozeni et al. [34]. Their encouraging results show that the host used could be competitive with the current *Haematococcus pluvialis* which is the main cultured organism for the industrial production of astaxanthin.

ASX is used in fish feed. However, the high temperature of the extrusion technique, used for processing fish feed, affects its stability and antioxidant capacity [66]. The retention of ASX is usually around 92% if the temperature during the extrusion process is at 90 °C, but it decreases to 85% at 100 °C.

## 3. Astaxanthin in the Food Industry

Most of the ASX used in fish feed production comes from synthetic sources. This synthetic ASX indirectly becomes part of human food. Food producers use ASX for its antioxidative property and the coloring it gives to foods. Hence, using ASX in food production could be a potential health management plan. Different products can be used to ensure that enough ASX becomes a part of everyday intake, for example, dairy products, fruit drinks, soy products, and protein shakes. The naturally produced ASX should be preferred to achieve this goal because the naturally produced ASX has a higher bioavailability, and lower cost. 

On the other hand, chemically produced ASX uses petrochemical compounds resulting in damage to the environment [66]. However, several limitations make the use of natural in industry ASX difficult. For example, natural ASX is usually unstable and degrades under temperature and long-term storage. In addition, due to its highly conjugated and unsaturated structure ASX can be damaged during the production process and technical steps, i.e., light, heat, storage conditions, etc. Another problem with ASX is its low solubility in water, leading to a lower availability during intake. Therefore, it also forms an emulsion over the water similarly to other fatty acid compounds. 

The chemical characteristics of ASX and other carotenoids, as well as several dietary and non-dietary factors, all affect how well they are absorbed [67]. Researchers have looked at how several animal species, such as mice, rats, dogs, and humans, absorb ASX from various sources. A double-blind experimental study reported that 28 physically fit men received 250 g of wild or farm-raised salmon every day for 4 weeks (5 mg ASX per day). Plasma ASX concentrations plateaued at 39 nmol/L after 6 days of administration with wild salmon (3S, 3′S isomer), and at 52 nmol/L after the administration of farmed salmon (3R, 3′S). It is interesting to note that after ingesting salmon from an aquaculture farm, the plasma levels of ASX were considerably higher on days 3, 6, 10, and 14, but not on day 28. These findings point to a similarity between the ASX isomer pattern in human plasma and that of the consumed salmon. Furthermore, it appears that even when ASX is taken from several sources, maximal levels can be attained during the first week of consumption when ASX intake is persistent [68]. Carotenoids are lipid-soluble compounds, and dietary lipids have a beneficial impact on ASX absorption. When administered in an oil-based formulation, ASX seems to be absorbed at a greater rate. Eight male adult participants were given a single dosage of 40 mg of ASX in three distinct lipid-based formulations (n = 8 for each group) in open parallel research. These three lipid-based preparations improved the bioavailability of ASX, although the preparation with the greatest proportion of the hydrophilic synthetic surfactant showed the greatest bioavailability. Consequently, such findings imply that ASX should be taken along with dietary fats to enhance bioavailability [11,69]. Thorough investigations should aim to reproduce these findings in dosages equal to those indicated by the different organizations, including the EFSA and FDA (US Food and Drug Administration), given the limited number of people involved in these bioavailability trials.

Several methodologies have been proposed to enhance the bioavailability of ASX. One of the strategies relies on encapsulation, which raises another question concerning what kind of compound should be used for encapsulation. Polymeric compounds are generally preferred in this regard. Still, one has to be sure that the selected polymeric compound does not alter the chemical nature of ASX and is also biodegradable once consumed. Proteins are also known for their emulsification properties. Milk proteins could act as a potential emulsifier due to their natural way of production and known health benefits. Sodium caseinate is an example of a protein successfully used to increase the stability of an ASX nano-dispersion. Such dispersions are usually prepared with the help of the emulsification evaporation technique. Researchers managed to obtain an optimum dispersion for ASX by carrying out three passes through a homogenizer using a pressure of 30 MPa at 25 °C [12].

Acute and sub-chronic toxicity of the ASX-rich biomass of *Haematococcus pluvialis* has been studied in Wistar rats. It was found that the oral LD_50_ was more than 12 g/kg body weight and showed no adverse effects in either male or female rats. Based on their findings, the researchers concluded that the recommended doses of ASX as a dietary supplement should be 2–6 mg/day or 0.07–0.1 mg/kg/day for an average individual weighing 60 kg [70]. ASX obtained from *Haematococcus pluvialis* has been recommended in a 24 mg/day dose for no more than 30 days in Europe, Japan, and the USA [71].

Considering the safety issues, the allowed levels of ASX in food supplements were up to 8 mg/day, and the acceptable daily intake for adults ranged from 0.034 to 0.2 mg ASX/kg body weight [28].

The consumption of ASX is beneficial not only for humans but also for animals. For example, the administration of 0.25 mg/kg body weight per day helped increase the milk yield and improve the health status of buffaloes [72]. Moreover, ASX also helped manage heat stress and inflation in egg-laying and broiler hens [73]. ASX also helped in combating heat stress in Karan and Sahiwal heifers [74].

Limited evidence in the literature devoted to showing improvements in ASX bioavailability reveals that this goal has not garnered significant attention. Novel delivery strategies, including various types of formulations, such as nanoparticles, topical application cream, and defined phospholipid complexes offer significant promise and are worthy of further exploration in attempts to enhance the bioavailability of this interesting beneficial molecule.

## 4. The Role of Astaxanthin in Managing Oxidative Stress

The imbalance of oxidation or the antioxidant mechanism in body cells facilitates the development of too many ROS and free radicals, resulting in oxidative stress. An essential mediator in the pathogenic development of illnesses is the increase in oxidative factors. These may interact with proteins, lipids, and DNA to cause protein inactivity, lipid oxidation, and damage to DNA in a chain reaction, which results in a wide range of disease conditions [75,76]. Cancer, cardiovascular disorders, autoimmune disease, ischemic disease, atherosclerosis, diabetes mellitus, and hypertension are the most common diseases caused by oxidative stress [77].

The role of ASX in the suppression of oxidative stress is significant. The antioxidant protection system becomes weaker with age. ROS associated with aging can be produced endogenously or exogenously, however, the mitochondrial ROS has the most prominent contribution to the aging process because mitochondrial dysfunction caused by oxidative stress is regarded as a key contributor to aging [78]. The body becomes more sensitive to oxidative stress and is prone to many diseases caused by the lack of antioxidant protection [79]. Several health concerns affecting seniors are mediated by oxidative stress and imbalances between pro-oxidants, such as ROS, and antioxidants, including the oxidation of blood lipids (cholesterol and triglyceride), increasing the risk of heart disease, pain, and stiffness in joints, cognitive decline, including mental awareness, information handling, and memory [13]. Research continues to validate the most effective ways to help reduce oxidative stress [2,80].

Therefore, ASX, being the most prominent antioxidant agent, is considered a source with active antioxidant properties and a distinctive nutritional supplement that can fight against oxidative stress and related damages to maintain health [56]. Dietary supplementation with ASX at any age can help combat oxidative stress and promote better health and well-being throughout life. 

Findings demonstrate that natural ASX is an extremely potent scavenger of ROS and a valuable ingredient for healthy aging formulations. It reduced oxidative stress in subjects and improved the serum lipid profile by normalizing serum triglycerides and increasing the levels of beneficial HDL-cholesterol [16]. The intake of ASX has been shown to prevent mitochondrial oxidative stress and improve the overall integrity of the mitochondrial membrane. This apparently leads to an increased energy generation capacity and improved cell energy status [16,17]. In addition, topical preparations containing ASX have been used in anti-aging formulations. A recent study has utilized lipo gel and hydrogel that contained ASX and other algal extracts for topical application [16]. 

After conducting 12 randomized clinical trials, including 380 participants, Ma and colleagues found that ASX could reduce the levels of biomarkers of oxidative stress and inflammation. Its intake decreased the concentration of blood malondialdehyde, improved the superoxide dismutase activity, and reduced serum isoprostane concentration in overweight patients [81]. 

A recent in vitro study has reported that the supplementation of 20 µM ASX on MCF-7 cells showed considerable pro-oxidant activity with a 53.3% increase in ROS in comparison to a 17.3% increase in the control. Findings of this study also mentioned that this effect improved (68.1% increase in ROS) the synergistic treatment of cells with a mixture of ASX, β-carotene, and lutein [82]. These results indicate that ASX, despite its well-known antioxidant properties that protect cells against oxidative damage, may potentially cause oxidative stress in cancer cells [83,84].

## 5. The Role of Astaxanthin in the Aging Process

Aging in humans is a dynamic and progressive phenomenon that is accompanied by numerous health challenges, varying from individual to individual due to several factors, including genetics, lifestyle choices, environmental factors, and life events [79]. The body’s antioxidant and repair processes become less effective with age. Premature aging is also closely linked to oxidative stress. Aging is typically accompanied by reduced cellular energy production and increased free radical production. This leads to the overloading of defense systems and oxidative damage. From a biological point of view, aging involves accumulating oxidative damage in cells and tissues. Maintaining a healthy lifestyle, along with a balanced and nutritious diet, is linked to healthy aging and prolonged periods of better health [2]. As a result, there is a growing need for healthy items that are appropriate for the elderly population. Effective antioxidants could help in promoting healthy aging [85].

ASX could be regarded as a highly promising candidate geroprotector [23]. It efficiently protects the mitochondrial double membrane system to improve its efficiency in energy production [86].

Younger people are naturally better protected from free radicals and others toxins through the balanced activity of the mitochondria, efficient antioxidant and DNA repair systems, and active protein degradation machinery. ROS, otherwise known as pro-oxidants, are formed as by-products of a normal metabolism in our bodies when food is converted into energy. The mitochondrial respiratory chain is also one of the major sources of cellular ROS generation. Immune cells fighting bacterial infections also release ROS. High levels of ROS can initiate harmful alterations in key biomolecules, such as lipids, proteins, and DNA [13,87]. 

A recent study has investigated the anti-aging effects of ASX in the accelerated aging model [18]. The research used a combination of D-galactose (galactopyranose having D-form) and jet lag to induce aging in the mice model, and 0.01% ASX was administered in one of the groups to study the anti-aging effects. The results indicate that six weeks of ASX supplementation significantly prevent liver deterioration by stimulating D-galactose metabolism. Moreover, the antioxidant status and muscle functions improved in ASX-supplemented mice compared to the control group [18]. 

At the molecular level, ASX modulates several crucial cell-signaling pathways, such as JAK-STAT, NF-kB, and PPARγ pathways. In brief, ASX is a promising nutraceutical supplement for treating various health issues, such as hair loss, where inflammation and oxidative stress play a critical role in the onset and subsequent progression of the health issue. ASX helped improve cognitive function in healthy, aged individuals. A human trial (n = 44) that evaluated ASX supplementation with a 12 mg daily dose for 12 weeks suggested that ASX may help protect against age-related cognitive decline [88]. A randomized clinical trial was carried out involving 32 healthy human participants between 60–70 years of age with confirmed signs of oxidative stress to evaluate the effect of ASX on aging. This study demonstrated that the supplementation of a lysosomal formulation of dark chocolate, having 7 mg of co-crystalized ASX with enhanced bioavailability, displayed fascinating effects on the improvement in oxidative status in aging human participants. It suggested the potential benefits of a combination of ASX with dark chocolate [89].

### 5.1. The Role of Astaxanthin in Skin Aging

ASX supports normal healthy skin by improving skin elasticity and moisture and reducing wrinkle formation (Table 2). ASX has been shown to have anti-inflammatory, immune-modulating, and DNA repair properties, which can effectively maintain skin health [90].

It was observed that the ASX supplementation of 6 or 12 mg prevented the secretion of inflammatory cytokine from keratinocytes and reduced the secretion of matrix metalloproteinase-1 by dermal fibroblast, thus preventing skin damage and helping in the maintenance of healthy skin in participants [95]. Another study has demonstrated that ASX reduced the transepidermal water loss attributed to ultraviolet exposure by decreasing the expression of aquaporin 3 and other proteins, thus reducing skin damage [91].

ASX supplementation (4 mg/day) for four weeks has been shown to rejuvenate the skin by reducing lipid oxidation and corneocyte desquamation in subjects above 40. The promising effects of ASX were mainly attributed to its antioxidant properties [99].

Human studies showed that 6 mg/day of ASX for six to eight weeks might reduce wrinkles, water loss, and age spots. ASX also improved elasticity, moisture content, and skin texture, and the effects seem to be enhanced when combined with the application of ASX topically [11]. In a double-blind trial in Japanese subjects, 4 mg of ASX supplementation, reduced the skin damage caused by exposure to UV rays [96]. ASX supplementation significantly reduced skin damage and helped maintain skin moisture compared to the placebo group [11]. Skin changes include a loss of elasticity and the proper function of oil glands, thinning skin layers, and the accumulation of pigments. These and other factors cause wrinkles, age spots, and dry/loose/sagging skin. 

Chung et al. detected experimentally that ASX significantly inhibited the ultraviolet-induced cytotoxicity and cell death of epidermal keratinocytes [100]. The clinical studies support the benefits of ASX supplementation (3–6 mg/d) on photoaged skin [101]. The administration of ASX reduced UV-induced wrinkle formation and increased collagen fibers in the skin [92].

### 5.2. The Role of Astaxanthin in the Brain Aging

Brain aging is associated with decline in cognitive function and motility. As ASX is capable of crossing the blood-brain barrier and its intake could have a healing effect on brain aging [102]. Recent research has validated ASX’s ability to protect the central nervous system. Much of this research has been centered on the neuroprotective benefits of ASX (Table 2). The neuroprotective effects of ASX were rewieved by Fakhri et al. from a clinical perspective [103]. 

In this area, two human clinical trials were carried out in Japan [97,98]. The first study took ten elderly subjects with age-related forgetfulness and administered 12 mg of ASX each day for 12 weeks [98]. The researchers found efficacy for age-related decline in cognitive and psychomotor function. The second study was randomized, double-blind, and placebo-controlled: a study on human volunteers. After 12 weeks at either 6 mg or 12 mg of daily ASX, subjects were found to have decreased phospholipid hydroperoxides levels (which accumulate in people with dementia) and improved erythrocyte antioxidant status. The researchers concluded that ASX supplementation might contribute to the prevention of dementia in humans as they age [97]. Human brain cells were subjected to an oxidative stress-induced neuronal cell damage system at Nagoya University in Japan. Significant protection was found in cells pre-treated with ASX [104]. 

Additionally, pre-treatment with ASX inhibited the generation of ROS. The authors concluded that the neuroprotective effect of ASX depends upon its antioxidant potential and mitochondria protection; therefore, it is strongly suggested that treatment with ASX may be effective for oxidative stress-associated neurodegeneration and a potential candidate for natural brain food. ASX can protect against damage from ischemia, the condition where there is a deficient supply of blood to the brain due to an obstruction of the arteries, which results in stroke, brain cell death, and impaired brain function [105]. The researchers attributed ASX’s benefits to its intense antioxidant activity. Another study found that pretreatment with ASX for five hours, and again one hour before ischemia, protected against brain damage [93]. ASX was found to be a potent agent against neurodegenerative disorders. ASX reduced brain cell death. Lastly, ASX displayed an ability to improve the proliferation of neural stem cells. The flurry of activity in 2009 and 2010 was not the first research on ASX benefits for the brain; a series of tests on rodents before this at the International Research Center for Traditional Medicine in Japan showed ASX’s potential as a supplement for the brain [106]. In the first experiment, blood pressure was reduced by the introduction of ASX to hypertensive rats. Blood pressure is a causative factor for many eye and brain diseases. The researchers went on to examine the effects of ASX on stroke-prone rats. After five weeks of continuous supplementation, the stroke incidence was delayed in the treated group. Next, it was established that the possible mechanism for these in vitro findings is nitric oxide suppression [107,108].

The same study demonstrated a neuroprotective effect on ischemic mice. In this case, ischemia was induced by blocking the carotid artery [8]. In humans, this condition can be caused by plaque buildup, which can block blood flow through the carotid artery in the neck, the primary source of blood to the brain. This plaque buildup can lead to many types of dementia [94]. 

ASX can alleviate the adverse effects of homocysteine accumulation, glutamate excitotoxicity, and oxidative stress on neuronal cells [109]. ASX reduces neuronal deficits and protects the rat brain from oxidative damage due to ischemia-reperfusion injury [93].

## 6. Other Pharmacological Activities of Astaxanthin

ASX is a powerful anti-inflammatory and antioxidant molecule due to its role in maintaining the integrity of mitochondrial membranes [110]. The antioxidant properties of ASX have been actively studied. ASX was useful for improving the chronic inflammation process caused by lipopolysaccharide *Escherichia coli* O55, which affects the mucous membrane of the oral cavity [111].

Studies show that ASX helps balance the immune system and suppress overactive immune responses that can create inflammation [112]. ASX supports cardiovascular health by improving blood lipid profiles in healthy seniors. As a bioactive compound, ASX has beneficial health effects for humans in preventing degenerative syndromes, such as cancer and cardiovascular disease [113]. A high dose (≥20 mg/day) of ASX showed a significant antioxidant effect after a 3-week intervention [114].

The antioxidant activity of ASX is many times higher than other antioxidants, such as *β*-carotene or *α*-tocopherol [115]. This property explains its therapeutic effects on certain metabolic disorders. ASX has been demonstrated to improve glucose metabolism in diabetics [116]. The 8-week administration of ASX supplementation improved the glucose level and reduced visceral body fat mass [117]. The randomized clinical trial was performed to investigate the potential effects of ASX supplementation on lipid peroxidation, insulin sensitivity, and anthropometric indices in participants with type 2 diabetes mellitus. The results showed that ASX can improve lipid metabolism in humans [107].

ASX has a protective effect against cholesterol and triglyceride oxidation. ASX also helps boost mitochondrial energy delivery, which allows the heart muscle to contract more powerfully and efficiently [56,118]. Research indicates that most diseases associated with the brain result from oxidation and/or inflammation. Antioxidants that can cross the blood-brain barrier are essential for people to protect the brain and central nervous system as they age. The human body may lose the ability to produce high levels of the antioxidants that are normally synthesized internally, such as superoxide dismutase (SOD), catalase, and glutathione peroxidase. Additionally, the human body is now subjected to unprecedented levels of oxidation caused by environmental factors, such as pollution, widespread toxic metals, contaminants, processed food, and high levels of stress [119]. All of these lead to an assault on vital organs during aging, particularly in the brains and eyes [120,121]. ASX helped support eye health and protected the eyes by reducing oxidative damage and improving blood flow in capillaries. Studies of individuals with age-related macular degeneration have demonstrated significant improvements in retinal health when given ASX and other carotenoids [122]. Liposome-encapsulated ASX demonstrated a hepatoprotective effect in lipopolysaccharide-induced acute hepatotoxicity [123].

ASX is safe in human clinical trials, and its intake has been shown to reduce cellular DNA damage and pro-inflammatory milieu [16].

The involvement of oxidative stress in antimicrobial therapy has remained an important issue over the years [124]. Significant antibacterial activity of crude ASX extract obtained from *Haematococcus pluvialis* was found against *Escherichia coli*, *Salmonella typhi*, *Vibrio cholera,* and *Staphylococcus aureus* by Rather and coworkers [125]. Results of this study showed that 10 µL of ASX extracted from *Haematococcus pluvialis* has the highest antibacterial potential (10.2 ± 0.20 mm) against *Escherichia coli*, while having the least antibacterial potential (6.1 ± 0.0 mm) against *Vibrio cholera* [125].

A global pandemic on novel coronavirus COVID-19 leads to severe morbidity and mortality worldwide. SARS-CoV-2 causes elevated levels of inflammatory factors, including interleukin-6 and tumor necrosis factor-alpha [112]. Subjects with comorbidities showed an increased risk of acute disease prognosis and of developing severe symptoms [126]. Scientists and therapists are searching for effective antiviral, anti-inflammatory, and antioxidative agents that would be useful in preventing the progression of COVID-19 [127,128]. ASX demonstrates great potential in reducing complications of COVID-19, considering its antioxidant, anti-inflammatory, autophagy-modulatory, and anti-apoptosis activities [112,129].

## 7. Concluding Remarks

**Figure 2:**
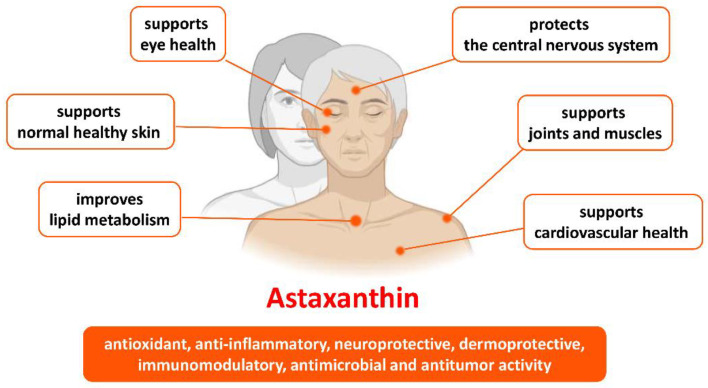
Benefits of astaxanthin in health and age-related conditions.

ASX is a xanthophyll reddish-orange carotenoid that shows significant biomedical applications. It is synthesized naturally by different living organisms, such as microalgae, fungi, lichens, and bacteria; it can also be produced biotechnologically. Besides, the reddish flesh of some animals (salmon, shrimps, lobsters, crayfish, etc.) is due to feeding on the ASX-producing organisms. Oxidative stress is a key contributor to several diseases, including aging and age-related disease. The significant antioxidant, anti-inflammatory, neuroprotective, skin-protective, immunomodulator, antimicrobial, and anticancer activity, as well as the ability to improve lipid metabolism, make ASX a promising compound for the prevention or even treatment of different health conditions (Figure 2). An additional important role of ASX has been reported, i.e., suppressing the development of lifestyle-related diseases, such as diabetes. Strong evidence shows that ASX holds great promise for those wishing to prevent cognitive diseases and maintain general brain health. The implications of the studies cited above are extremely exciting, as the proportion of the elderly and the number of patients with cognitive decline increase in the population. Researchers validated the significant benefits of ASX supplementation for healthy aging. Consequently, the demand and research for natural ASX for human health are increasing extensively worldwide. This review highlighted important ASX-associated clinical trials and explored many clues for research on the nutritional aspects of healthy ASX to learn much more about its value for healthy aging and for the management of age-related disorders.

## Figures and Tables

**Table 1:** Diversity of the natural sources of ASX.

Group of Organisms	Representative	References
	*Haematococcus pluvialis*	[9,10]
Plantae (microalgae)	*Chlorella zofingiensis* *Chlorococcum* *Chromochloris* *zofingiensis* *Chlamydomonas reinhardtii* *Diatoms*	[33,34,35]
Fungus (yeasts)	*Xanthophyllomyces dendrorhous* (*Phaffia rhodozyma)* *Yarrowia lipolytica ^§^* *Saccharomyces cerevisiae ^§^*	[36,37,38]
Lichene	*Clodia aggregata,* *Concamerella fistulata,* *Usnea amaliae*, *Usnea densirostra*	[39]
Bacteria	*Corynebacterium glutamicum* *^§^* *Cyanobacteria* *(Synechococcus sp.)* *Agrobacterium aurantiacum* *Paracoccus carotinifaciens* *Escherichia coli ^§^*	[38,40,41]
Animalia	Redfish Crustaceans (*Euphausia superba,* *Pandalus borealis, Calanus finmarchicus, etc*.) Wild salmon (*Oncorhynchus* species)	[4,7,26]

^§^ Genetically modified organism (GMO).

**Table 2:** Summary of some pre-clinical and clinical studies evaluating potential role of ASX in the management of aging, including skin and brain aging.

Routes of Administration	Concentrations	Experimental Model	Goals/ Health Benefits	Reference
Oral	0.25 mg/kg BW/day	Buffaloes	Increase of milk production and improvement of overall health	[72]
Oral	10, 20, 40, and 80 mg/kg	Broiler hens	Management of heat stress and inflammation	[73]
Oral	0.25 mg/kg BW/day	Heifers	Prevention of heat stress	[54]
Oral	0.01% ASX	Mice	Improvement of the oxidative status and muscle function	[18]
Topical	20 J/cm^2^	Mice	Prevention of photoaging caused by UV irradiation	[91,92]
Oral	25 mg/kg	Rats	Protection from oxidative damage caused by cerebral ischemia-reperfusion injury	[93]
Transcutaneous intrathecal (i.t.) injection	10 μL of 0.2 mM	Rats	Protection against spinal cord injury-induced neuronal loss, demyelination, and functional deficit	[94]
Oral	5 mg per/day	Human	Study of the bioavailability of ASX	[68]
Oral	40 mg	Human	Study of the bioavailability of ASX	[69]
Oral	12 mg/day	Human	Prevention age-related cognitive decline	[88]
Oral	7 mg/day	Human	Improvement of the oxidative status	[89]
Oral	6 mg or 12 mg	Human	Prevention of age-related skin damage and improvement of skin conditions	[95]
Oral	4 mg/day	Human	A strong antioxidant effect and facial skin rejuvenation	[46]
Oral	4 mg	Human	Reduction the skin damage caused by exposure to UV rays	[96]
Oral	6 or 12 mg/day	Human	Prevention of age-related dementia	[97,98]

## Data Availability

Not applicable.
